# Modulation of tau phosphorylation by environmental copper

**DOI:** 10.1186/2047-9158-3-24

**Published:** 2014-11-17

**Authors:** Kellen Voss, Christopher Harris, Martina Ralle, Megan Duffy, Charles Murchison, Joseph F Quinn

**Affiliations:** Department of Neurology and Parkinson’s Disease Research Education and Clinical Care Center (PADRECC), Portland Veterans Affairs Medical Center, Portland, OR USA; Department of Neurology, Oregon Health and Sciences University, 3181 SW Sam Jackson Park Road, Portland, OR 97201 USA; Department of Molecular and Medical Genetics, Oregon Health and Sciences University, Portland, OR USA

**Keywords:** Copper, Alzheimer’s disease, Tau protein, Transgenic mice

## Abstract

**Background:**

The transition metal copper enhances amyloid β aggregation and neurotoxicity, and in models of concomitant amyloid and tau pathology, copper also promotes tau aggregation. Since it is not clear if the effects of environmental copper upon tau pathology are dependent on the presence of pathological amyloid β, we tested the effects of copper overload and complexing in disease models which lack pathological amyloid β.

**Methods:**

We used cell culture and transgenic murine models to test the effects of environmental copper on tau phosphorylation. We used oral zinc acetate as a copper lowering agent in mice and examined changes in blood and brain metals through inductively coupled plasma mass spectroscopy. Behavioral effects of copper lowering were assessed with Morris water maze and novel object recognition tasks. Changes in tau phosphorylation were examined by phosphorylation specific antibodies on Western blots.

**Results:**

In human neuroblastoma cells, excess copper promoted tau phosphorylation and a copper complexing agent, tetrathiomolybdate, attenuated tau phosphorylation. In a transgenic mouse model expressing wild type human tau, copper-lowering by oral zinc suppressed plasma and brain levels of copper, and resulted in a marked attenuation of tau phosphorylation. No significant changes in behavior were observed with copper lowering, but a trend to improved recognition of the novel object was observed in zinc acetate treated mice.

**Conclusions:**

We propose that reduction of brain copper by blocking uptake of copper from the diet may be a viable strategy for modulating tau pathology in Alzheimer’s disease. The potential benefits of this approach are tempered by the absence of a behavioral benefit and by the health risks of excessive lowering of copper.

**Electronic supplementary material:**

The online version of this article (doi:10.1186/2047-9158-3-24) contains supplementary material, which is available to authorized users.

## Background

Alzheimer’s disease (AD) is a progressive neurodegenerative disease that manifests symptoms years after initial abnormal processes begin. It is characterized pathologically by the presence of extracellular amyloid β (Aβ) plaques and intracellular neurofibrillary tau tangles (NFTs). The largest risk factor for AD is age, however increasing evidence points to other environmental factors as playing a role in AD. In particular, the transition metals zinc and copper have been shown to be involved in the progression of AD. Studies examining metal levels in humans indicate an increase of copper during aging, and further increased concentration of copper within AD brains [1]. Aβ has been shown to bind copper both in vitro and in vivo [2]. The binding of copper induces spontaneous aggregation at rates faster than in the absence of the metals [3–6]. When bound to Aβ, copper can undergo redox reactions and generate reactive oxygen species extracellularly [7, 8]. As well, copper is found concentrated in NFTs [9], can bind to tau protein [10–12], and accelerates aggregation rates in vitro [13]. Previous studies examining the effects of chronic copper exposure in 3×Tg mice (APPswe, PS1, P301L tau), showed that copper increases plaque formation and enhances tau phosphorylation [4].

Previous studies examining various copper lowering therapies have attempted to chelate copper from the brain. Clioquinol and its derivative PBT2 were developed as blood brain barrier permeable chelators and had promising results in clinical trials at improving cognition and Aβ in the CSF [3, 5, 6, 14, 15]. Tetrathiomolybdate was previously utilized by our lab in Tg2576 mice to chelate copper from the brain, and showed reduced Aβ plaques in a prevention paradigm, however not in a treatment paradigm [16]. We recently published results utilizing the Tg2576 mouse model and the systemic copper lowering therapy, zinc acetate [17]. This treatment works by induced expression of metallothionein, resulting in a Cu-metallothionein complex which prevents uptake of copper in the gut. This treatment is utilized in humans with Wilson’s disease, a genetic disorder resulting in increased copper accumulation primarily in the liver, brain and cornea [18]. In our study we found that independent of genotype, oral zinc acetate administration was able to reduce both blood and brain copper levels while not affecting brain zinc levels [17]. This resulted in decreased brain and blood copper, and reduced Aβ insolubility in a prevention paradigm, but not a treatment paradigm. Another group has also examined the use of zinc sulfate in the 3×Tg mouse model and observed decreased Aβ and tau phosphorylation [19]. Whether the effect of copper on tau phosphorylation is independent of the Aβ/copper interaction is yet to be determined.

We tested the hypothesis that copper modulates tau phosphorylation in the absence of pathological Aβ in a human neuroblastoma cell line (SH-SY5Y) and in a transgenic mouse expressing wild type human tau (hTau). The SH-SY5Y cell line is a widely used model, including studies of tau biology. The hTau mouse model was selected due to the expression of full length wild-type human tau, which more closely resembles idiopathic AD. This mouse model contains the entire wild-type human tau gene with the endogenous mouse tau gene disrupted, resulting in hyperphosphorylated tau and accumulation in an age dependent manner.

## Materials and methods

### SH-SY5Y cell culture

The human neuroblastoma SH-SY5Y cells were obtained from ATCC (CRL-2266) and grown in DMEM/F12 Media (11320–082 Gibco) and supplemented with 10% FBS (16000–044 Gibco), non-essential amino acids (NEAA) (11140–050 Gibco), and penicillin/streptomycin (15140–122 Gibco). For experiments, cells were plated in growth media with 10 μM retinoic acid (R2625 Sigma) (in DMSO) to induce outgrowth of projections and upregulation of tau protein [20–22]. Copper was added at concentrations up to 400 μM (in water), based upon dosing studies to find a robust induction of tau phosphorylation, and Tetrathiomolybdate (323446 Sigma) was added at 3–90 μM (in DMSO). Cells were harvested by scraping, followed by centrifugation, rinsing in PBS, and homogenization by sonication in the same extraction buffer used for brain homogenization (see below). Protein concentrations were determined by BCA kit (23227 Pierce).

### Animals and diet

The hTau strain is a double transgenic mouse crossed between the 8c (endogenous mouse tau with eGFP inserted in exon 1) and Mapt (full length human MAPT that expresses all six splicing isoforms on human promoter). Mice were generated from a breeding pair generously provided by Dr. Glen Kisby. Male mice hemizygous for the human microtubule-associated protein transgene were harem bred with female murine microtubule-associated protein knockouts. After weaning and genotyping, litters were housed by sex with 4 mice per cage. Mice were housed in a 12-hr light/12-hr dark cycle, and ad libitum fed AIN-93M Purified Rodent Diet (Dyets Inc., Bethlehem, PA) and water. We have previously used the AIN-93M Purified Rodent Diet as a maintenance diet to control the amount of baseline cupric carbonate (6 mg/kg), zinc carbonate (30 mg/kg), and other minerals and vitamins [23]. 70 g/kg of soybean oil, containing 7% of an omega-3 fatty acid called linolenic acid, is present in AIN-93M. The NIH Guidelines for the Care and Use of Laboratory Animals were used for the procedures and the Institutional Animal Care and Use Committee of the Portland VA Medical Center approved this study (Protocol Number: 1409). All behavioral studies and handling were done to minimize animal suffering.

### Treatments

HTau mice were randomized to either normal or zinc acetate drinking water (zinc acetate from Mallinckrodt Baker, NJ). Animal weights and ceruloplasmin (Cp) activity were monitored to ensure zinc levels were not detrimental to the animal’s well-being. In all of the treatment cohorts, oral zinc acetate was administered at a dose of 2 g/L vehicle in their drinking water for the first four months, and then 1.5 g/L for the remaining two months. Cp activity confirmed that mice were below the target copper levels of 50% at 4 months, so the dose was reduced to prevent complications. Females received an effective dose of 176.59 mg Zn/kg mouse/day and males received an effective dose of 143.6 mg Zn/kg mouse/day (vehicle = DiH_2_O). Mice began receiving treatment at 12 months of age for a period of 6 months. At the end of six months mice underwent behavioral training and were euthanized.

### Behavioral analysis

Spatial learning and memory associated with the hippocampus was assessed using the Morris water maze (MWM) as described in our previous studies [16, 24, 25]. Briefly, mice were placed into the MWM tank for 6 trials each day for 7 days. The first two days were visual platform testing in which the platform was slightly above the water with a graduated cylinder on top. Platform location was rotated equally between each quadrant to prevent bias towards any given quadrant. Mice were given 60 seconds to find the platform before being placed back in their cage. Forty-eight hours after visible platform training, hidden platform training was conducted for five days. The target quadrant became the upper right quadrant and the platform was not moved during hidden platform training. The platform was slightly submerged in opaque water and mice were given 60 seconds to find the platform. On the last day of hidden platform training, the platform was removed from the tank and mice were given a probe trial 2 hours after the final training, in which they were allowed to swim freely in the pool for 60 seconds. Seventy-two hours following the first probe trial a second probe trial was conducted. The inter trial interval for each trial was 10 minutes, except for between trials 3 and 4 which were 2 hours and correspond to the beginning of session one and session two for each day. All MWM tasks were evaluated using an ANY-maze video tracking system. Performance on visible and hidden platform training was expressed as the mean ± standard error of the mean (SEM) of the latency to platform (in seconds). Performance on probe trials was expressed as the mean ± SEM of the percent time in target quadrant ((seconds in quadrant/total seconds)*100) for all mice in each treatment group.

### Tissue homogenization

Following behavioral testing, animals were euthanized according to the IACUC standard protocol of CO_2_ inhalation followed by cervical dislocation. Plasma was collected by centrifugation of blood, and frozen. The left hemisphere was utilized for analysis in this study. The frontal cortex slice (the anterior 2 mm) and frontal slice (the next 2 mm) were snap frozen on dry ice. The frontal slice was used for inductively coupled plasma mass spectroscopy (ICP-MS), described below. The remaining left hemisphere was divided into approximately equal anterior and posterior fractions and snap frozen. The left anterior portion of the brain was homogenized using an updated, previously published protocol for amyloid extraction [26]. Briefly, tissue was homogenized by sonication in buffer, termed AT+, containing: 10mM Tris pH 7.5, 1mM EGTA, 1mM DTT, 10% Sucrose, 1% Triton X-100, 1× protease inhibitor cocktail (Sigma P8340), 1× phosphatase inhibitor cocktail (Sigma P0044). Homogenates were centrifuged in a TLA-100 fixed-angle rotor at 393,000 × g for 20 minutes at 4°C a total of three times. After each spin, supernatants were snap frozen on dry ice and pellets were re-sonicated in original AT + buffer. Following the three spins, the pellets were washed with water and again centrifuged. Following this wash, the pellet was sonicated in 70% formic acid to solubilize amyloid protein and a final centrifugation step was performed.

### Ceruloplasmin assay

Blood was collected at baseline for every animal and then every 4 weeks from 6 animals per treatment group, rotated equally between animals. Up to 200 μl was obtained from the saphenous vein. Cp activity was measured from the beginning and end of treatment using a modified version of the oxidase method that we have previously reported [16]. Data was expressed as the mean ± SEM.

### Analysis of brain and plasma metals

Brain tissue (frontal slice) and plasma was analyzed for zinc and copper levels using ICP-MS, following a previously used protocol [17]. Briefly for brain tissue, approximately 75 mg of tissue from male and female mice was digested in 750 μl of concentrated HNO_3_ at 90°C for 1 hour. Once cooled, 750μl hydrogen peroxide was added to each tube and 750μl 1% HNO_3_ was used to rinse the sides. Briefly for plasma metals, 40 μl of plasma was diluted 51× in 1% HNO_3_. ICP-MS was performed using the previously reported parameters and standards [17]. Samples were analyzed in triplicate and averaged.

### Western blotting

SH-SY5Y and murine brain homogenates were analyzed for various proteins through Western blotting. Proteins were separated by SDS-PAGE with 10 μg per lane for SH-SY5Y homogenates and 15μg for murine homogenates. Protein was transferred to PVDF membrane and blocked with 5% BSA. Primary (dilutions below) and secondary antibodies (1:2500 dilution) were incubated on blots in 0.5% BSA. Antibodies used in this study are:

AT8 [phosphorylated S202/T205 tau] (Thermo) 1:500 mouse IgG, AT180 [phosphorylated T231/S235 tau] (Thermo) 1:500 mouse IgG, PHF-1 [phosphorylated S396/S404 tau] (Peter Davies) 1:500 mouse IgG, pT231 [phosphorylated T231 tau] (Thermo) 1:1000 rabbit IgG, Tau12 (Skip Binder) 1:5000 mouse IgG, GSK-3β total (Cell Signal) 1:1000 mouse IgG, Phosphorylated-GSK-3β S9 (Cell Signal) 1:1000 rabbit IgG, CDK5 (Cell Signal) 1:1000 rabbit IgG, p35/p25 (Cell Signal) 1:500 rabbit IgG, and GAPDH (Thermo) 1:5000 mouse IgG. Blots were imaged using a Bio-Rad Gel Doc XR Imaging platform with Image Lab software. Images were exported as *.tiff, quantified using Image J, and assembled using Adobe Illustrator.

### Statistics

For the MWM and NORT, data was plotted using GraphPad Prism 5 software (GraphPad software Inc.) and analyzed using both GraphPad Prism and R 3.0 (R Foundation for Statistical Computing) as previously described [17]. Data for Western blotting was analyzed through Prism using a one-sided student’s t-test for significance (p < 0.05).

## Results

### Copper enhances tau phosphorylation independent of Aβ in neuroblastoma cells

**Figure 1:**
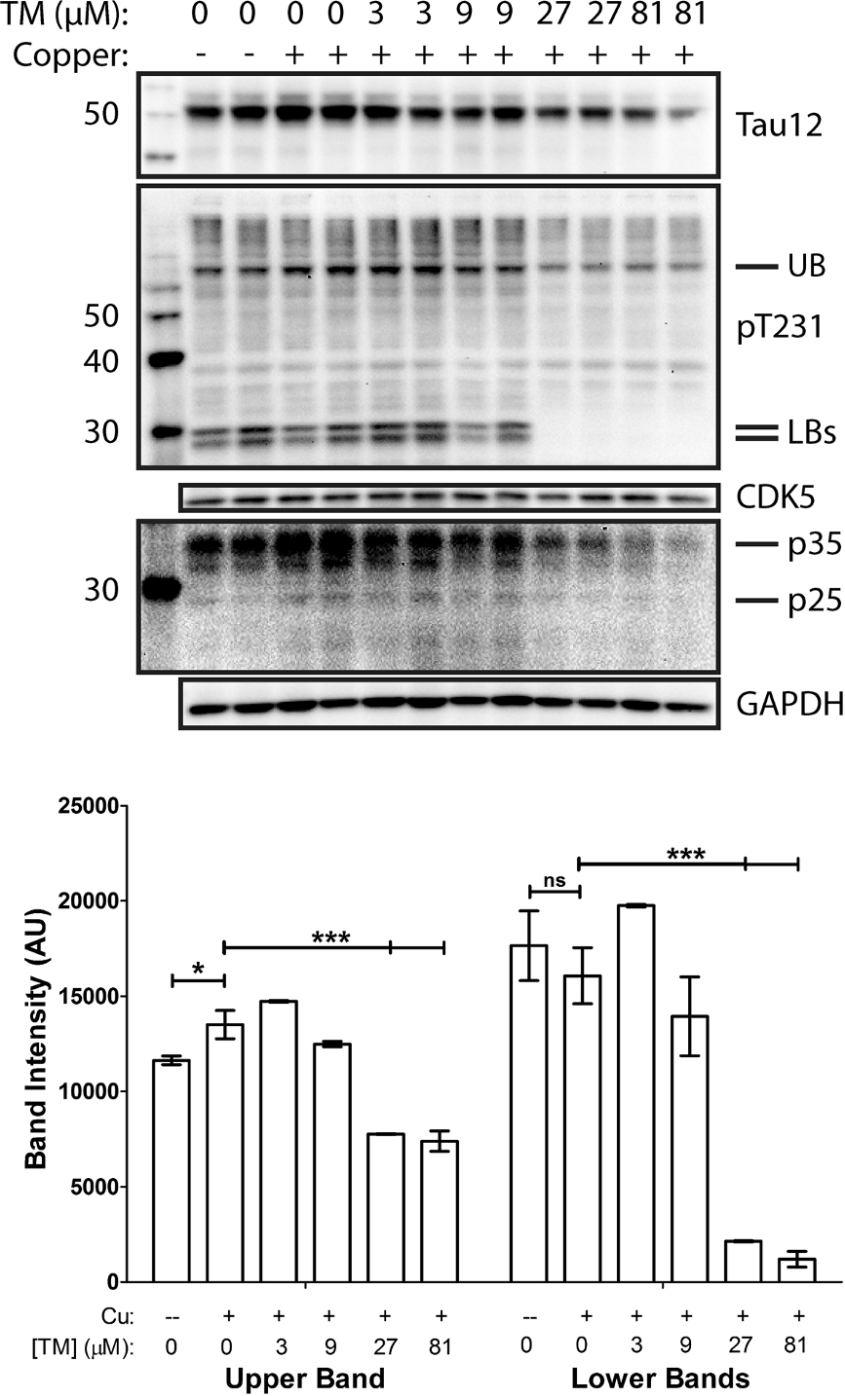
**Copper affects tau phosphorylation.** Varied copper and tetrathiomolybdate concentrations were added to the media of SH-SY5Y cells in an effort to elucidate the effects of copper on tau phosphorylation, with phosphorylation induced with copper at a concentration of 400 uM but not lower concentrations. Western blot bands for phosphorylated tau at T231 were quantified. The mean ± the SEM of the band intensity is represented. * = p <0.05, ** = p <0.01, *** = p <0.001.

Based upon previously published results [4, 17], we sought to determine if copper was able to induce tau phosphorylation in the absence of Aβ, indicating a potential direct and independent effect. Cells incubated in the presence of 400 uM copper for 24 hours had higher amounts of phosphorylated tau at an AD relevant site as compared to the cells incubated in the absence of copper (Figure 1). Lower concentrations of copper were ineffective, and phosphorylation of tau at other AD sites was less robust (data not shown). Incubation of cells with the copper complexing agent tetrathiomolybdate (TM) attenuated copper-induced tau phosphorylation in a dose-dependent manner (Figure 1). We also examined the involvement of CDK5, a kinase highly involved in tau hyperphosphorylation in AD and previously found to be affected by chronic copper exposure [4]. We found that the overall levels of CDK5 did not change with TM treatment, but levels of the CDK5 activators p35/p25 were reduced with increasing TM (Figure 1 and Additional file 1). This shows that TM complexing copper reduces the interaction of p35 and p25 (the neurotoxic cleaved form of p35), leading to reduced tau phosphorylation. Additionally cellular morphology and viability looked normal (data not shown).

### Zinc Acetate affects the levels of plasma ceruloplasmin and plasma and brain copper, but not brain zinc levels, in mice expressing wild type human tau

**Figure 2:**
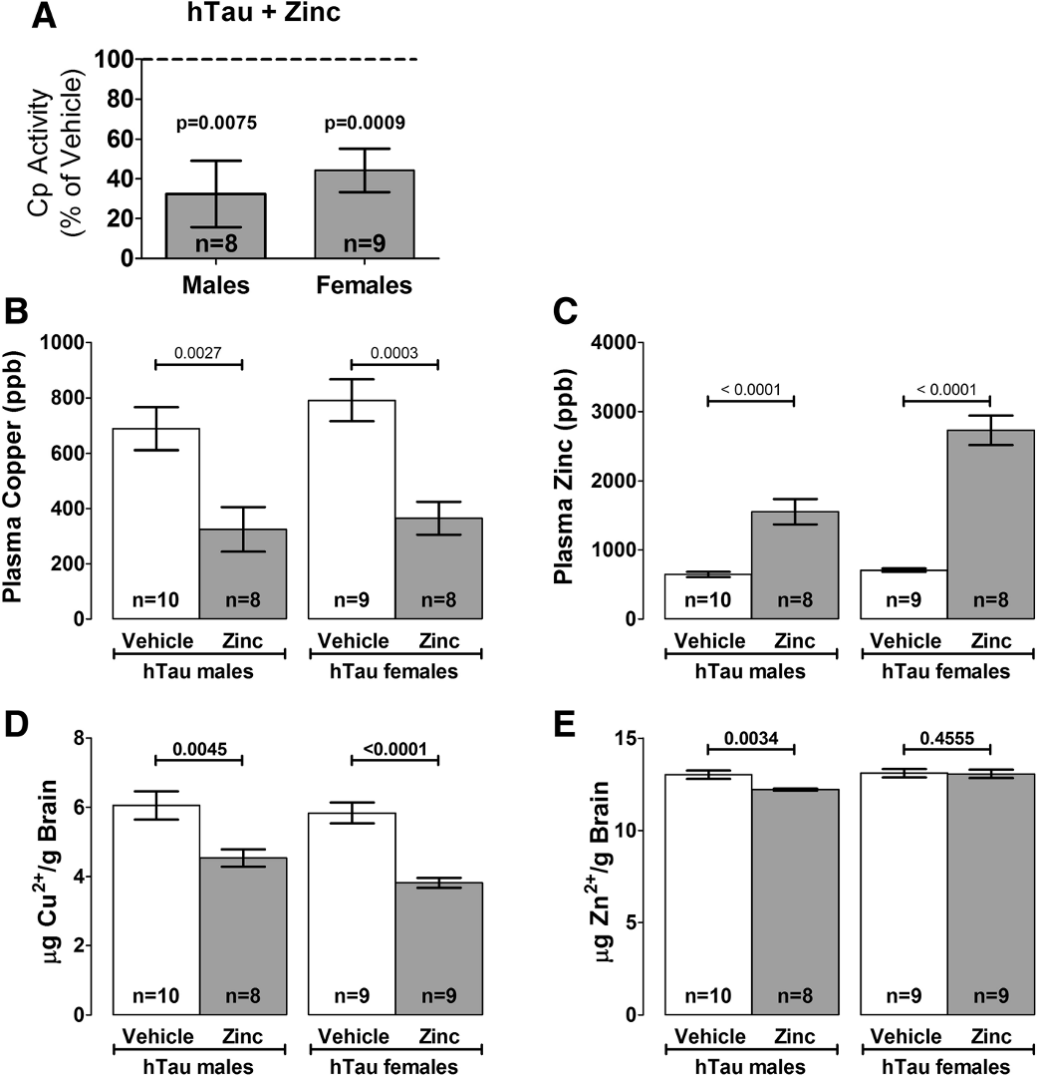
**Metal levels in hTau mice after six months of treatment. (A)** Copper levels in the plasma were determined by a surrogate, ceruloplasmin, for both males and females and plotted as a percentage of the vehicle treated mice. ICP-MS was used to analyze levels of copper and zinc in the plasma (**B** and **C** respectively) and brain (**D** and **E** respectively). Metal levels are plotted as parts per billion (ppb) for plasma and μg of metal per gram of homogenized brain tissue. Data is represented as the mean ± SEM of all mice in the treatment group.

The major copper carrying enzyme in the blood is ceruloplasmin (Cp). Cp expression is regulated by systemic copper, therefore, plasma Cp can be used as a surrogate marker of copper status. We found a marked reduction in plasma Cp activity in zinc treated male and female mice as compared to untreated mice. We further analyzed the brain metal composition of both copper and zinc using ICP-MS to determine if systemic copper lowering produced lower brain copper levels and unchanged zinc levels, as we have previously shown in other murine models [17]. We found a significant reduction in brain copper levels in zinc treated mice as compared to untreated mice (Figure 2). Importantly, brain zinc levels were not increased by oral zinc administration, and paradoxically, oral zinc produced a slight, but significant lowering of brain zinc in male mice. We also examined iron levels to determine non-specific effects and found no changes in plasma or brain iron (Additional file 2).

### Lowering of brain copper results in decreased tau phosphorylation

**Figure 3:**
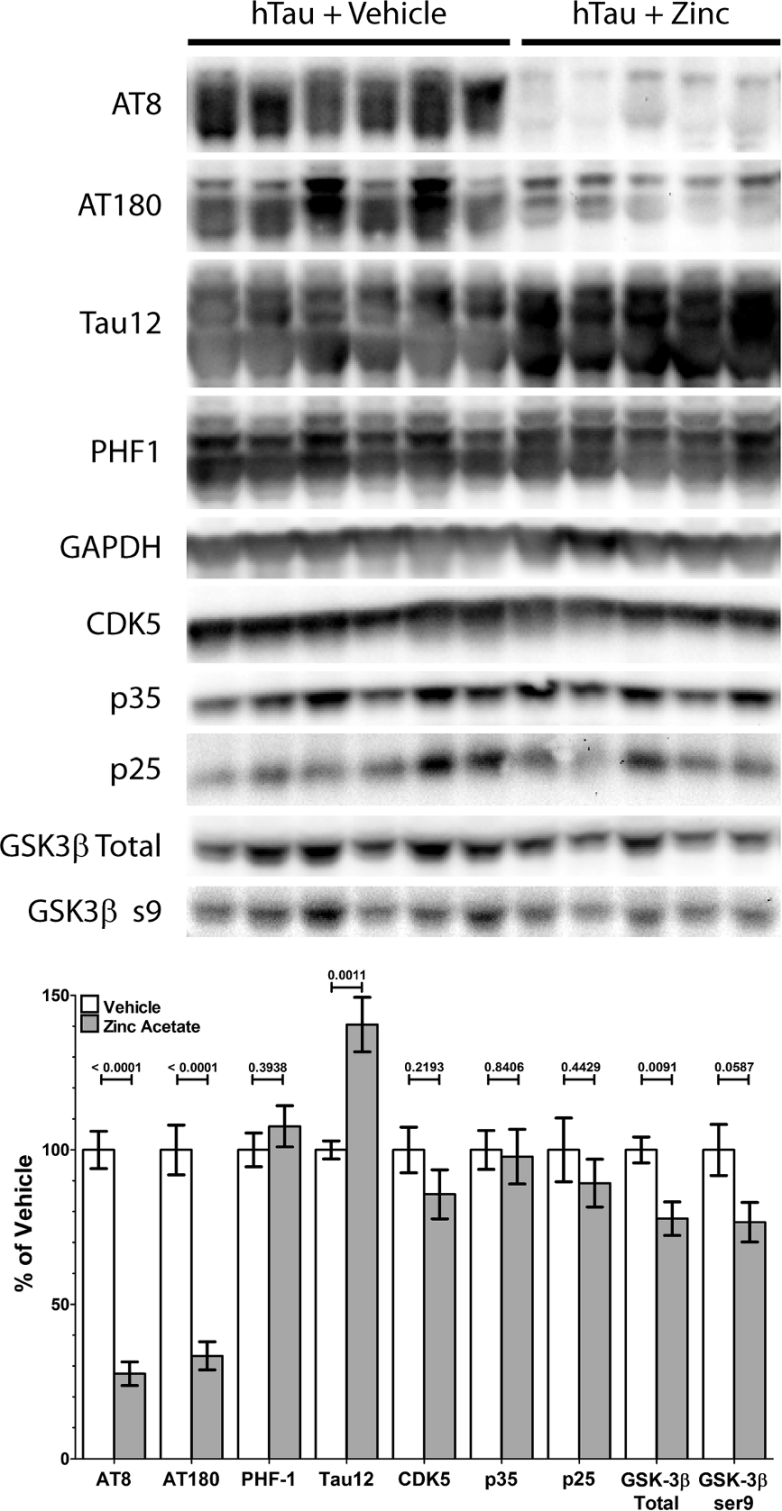
**Analysis of tau phosphorylation following zinc acetate treatment.** The soluble fraction of anterior left hemisphere homogenates for male mice was analyzed for AT8 (202/205), AT180 (231), PHF1 (396/404), Tau12 (total tau), CDK5, p35, p25, Total GSK-3β, and GSK-3βs9 (active) reactivity. Bands were quantified and plotted as percentage mean ± SEM of the vehicle treated mice for all mice in each treatment group.

Our proposed hypothesis was that a reduction in brain copper levels would affect tau phosphorylation, as was shown in previous studies and in our cell culture model. We analyzed tau phosphorylation through Western blots using site specific anti-phosphorylated tau antibodies (Figure 3). We found that in male hTau mice treated with zinc acetate, the phosphorylation state of tau at S202/T205 (AT8) and T231 (AT180) was significantly reduced as compared to hTau mice that were untreated. Phosphorylation at S396/S404 (PHF-1) did not seem to be affected by zinc acetate. Interestingly, we found that the total amount of tau was increased when brain copper was lowered. After adjusting for the ratio of pTau to total tau, AT8, AT180 and PHF-1 were found to be reduced with zinc treatment (Additional file 3). Female mice showed highly variable reactivity to all phosphorylated tau antibodies regardless of treatment group (data not shown). Additionally, we examined insoluble tau by a total tau Western blot of the formic acid soluble and Triton X-100 insoluble proteins and found no difference between treatment groups (data not shown).

There are many kinases known to phosphorylate tau at the sites reported here, however CDK5 and GSK-3β have been previously examined in relation to chronic copper exposure [4]. We examined the levels and activity of CDK5, previously reported to play a role during chronic copper exposure, and GSK-3β, not previously shown to play a role during chronic copper exposure. We found in male hTau mice, the overall levels of CDK5 and the levels of the regulator p35/p25 were not significantly affected by zinc acetate treatment (Figure 3). However, in male hTau mice we observed both total levels of GSK-3β and the inhibited form, phosphorylation at serine 9, decreased significantly with zinc acetate treatment (Figure 3). While the reduction of total and S9 phosphorylated GSK-3β were comparable, this data suggests that copper lowering therapy may affect the availability of GSK-3β, potentially reducing the likelihood of tau phosphorylation.

### Learning and memory testing was unaffected by zinc acetate

**Figure 4:**
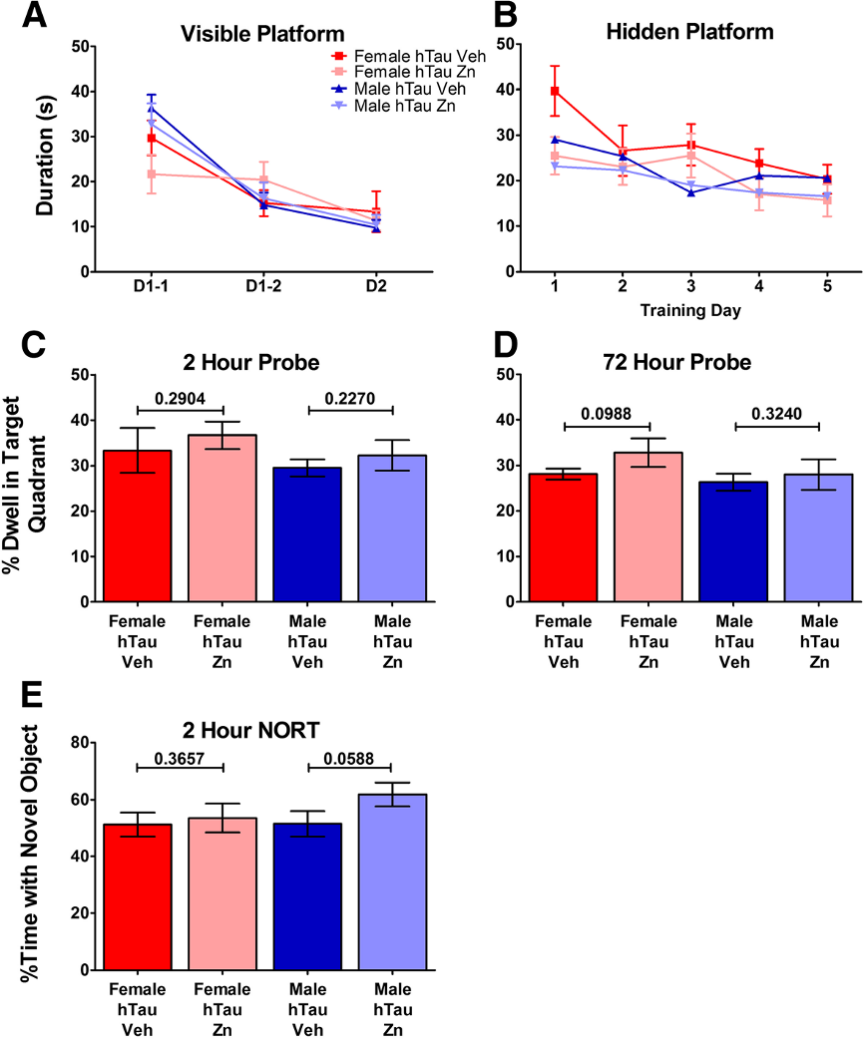
**Memory and spatial learning evaluation following zinc acetate treatment. (A)** Mice were trained for two days using a visual platform raised above the water level. Data represents the mean ± SEM of the time to reach the platform in seconds, for all mice in the treatment group. **(B)** The water level was raised and mice were trained to find the hidden platform using visual cues around the room. Each data point is the average of six trials per day per mouse in each treatment group and represented as the mean ± SEM of the time to reach the platform in seconds. Following hidden platform training, the mice were tested at **(C)** 2 hours and **(D)** 72 hours for the ability to remember where the hidden platform was located. Data is presented as the mean ± SEM of the percentage of time spent in the target quadrant for all mice in the treatment group. **(E)** Mice were analyzed for time spent with a novel object as a function of working memory. Data is represented as the mean ± SEM of the percentage of total time spent with the novel object for all mice in each treatment group.

In our previous study, we observed that despite a reduction in insoluble Aβ, there was no appreciable difference in the learning and memory between control and zinc treated mice. However, Aβ load correlates less well to cognition in humans, as compared to tau aggregation. We first analyzed our hTau mice utilizing the Morris Water Maze where we found no differences in swim speed between genders or treatment groups (data not shown). No significant difference was observed in learning by either visual platform or hidden platform in the hTau mice, regardless of gender (Figure 4A, B). When probed at 2 or 72 hours, we also failed to see a difference in the target quadrant dwell time between zinc and untreated mice, regardless of gender (Figure 4C, D). Additionally, we examined the number of crossings of the platform location and found no difference between genders or treatment groups (data not shown). We also assessed memory through the novel object recognition test. Male mice treated with zinc acetate had a longer amount of time spent with the novel object as compared to the untreated mice (62% versus 51%), indicating improved memory (Figure 4E).

## Discussion

These studies demonstrate that copper modulates tau phosphorylation in the absence of pathological Aβ. This is distinct from previous studies showing effects of copper upon Aβ pathology or upon concomitant Aβ and tau pathology [4, 17, 19, 27], and so represents an additional reason to consider copper-modulating strategies for the treatment or prevention of neurodegenerative diseases involving tau pathology, including AD. The phosphorylation sites affected are disease-associated phosphorylation sites which have been identified as candidate targets for the prevention of AD. The relevance to human subjects with AD is strengthened by the use of cell lines and animal models which express wild type human tau, the gene expressed in human AD. Importantly, zinc treatment did not cause an increase in brain zinc confirming our prior experience and that of other laboratories [17]. This is important as zinc also has been shown to affect tau phosphorylation [28–31]. The marked reduction of tau phosphorylation with brain copper reduction, with no increase in brain zinc, indicates a copper dependent rather than a zinc dependent mechanism for affecting tau phosphorylation in the brain.

At the same time, we recognize that these model systems are not ideal in other respects. Differentiated neuroblastoma cells are not fully differentiated neurons, and there is no functional change in these cells to indicate any consequences of tau phosphorylation. Additional experiments with primary neurons will permit the assessment of copper manipulations upon relevant markers of neuronal health, such as dendritic complexity and spine number.

The concentration of copper required to induce tau phosphorylation in vitro is also a relative weakness, as this relatively high concentration may not be physiologically relevant and may represent a non-specific stressor (Additional file 4). On the other hand, the 400 uM concentration employed here is within range of the approximately 100 μM concentration reported for human brain [32]. Reports of 4 fold increased copper in the neuropil of AD brains [32] and 2–15 fold increased copper in plaques [33] may help justify the use of very high copper concentrations in these experiments. However, we also note that our measurements of metals in brain tissue fail to distinguish between intra- and extra-cellular levels, and also fail to assess bioavailability of metals. Since other investigators have argued that intraneuronal levels of copper are specifically reduced in AD, our “bulk” brain levels of copper may be misleading. In fact, those investigators have presented evidence to suggest that delivering more, rather than less, copper to CNS neurons is the key to attenuating tau pathology [34]. Additional research will be necessary to resolve these apparent discrepancies.

The hTau mice also have some limitations. They do not develop neurofibrillary tangles or display neuronal death, so the histological consequences of copper modulation and tau phosphorylation are not evident in the animal experiments presented here. We attempted to assess the functional effects of these manipulations in the mice by looking for changes in cognitive abilities. Although there was no evidence of any treatment effect upon hippocampal-dependent memory (Morris Water Maze), there was a trend toward treatment efficacy upon frontal cortex-dependent memory (Novel Object). We used an hTau-/- and mouse tau (mTau) -/- strain to compare behavioral changes of the mTau-/-, hTau+/+ strain. A more appropriate control for behavioral comparisons may have been the mTau+/+, hTau -/- strain. However, the mice used in this study were still able to learn the tasks, suggesting that they are suitable for detecting symptomatic effects. Additional experiments in mice with more robust tau pathology, neuronal damage, and behavioral effects are under way and will help clarify whether the observed effect on tau phosphorylation is functionally relevant.

Another limitation of this strategy is the health risks of lowering systemic copper levels. There are both hematologic and neurologic complications of copper deficiency which will require close monitoring if this copper lowering approach is applied clinically. The dosage used in mice in these experiments is based on previous dose finding work to achieve copper lowering of 30-50%, which was well tolerated by the mice in terms of body weight and survival. Similarly, human subjects with Wilson’s disease tolerate oral zinc therapy and achieve a negative copper balance [35], so clinical application (with appropriate monitoring) of zinc therapy for AD is at least plausible.

Finally, the mechanism by which tau phosphorylation is modulated is not entirely clear, as the in vitro studies implicate one kinase involved in tau phosphorylation (CDK5) while the in vivo data implicate another (GSK-3β). Resolution of this point will require additional experimentation, but may not be necessary for decisions about translating this strategy to clinical situations in which modification of tau phosphorylation is desirable, regardless of the kinase involved.

## Conclusion

In summary, we present evidence that human wild type tau phosphorylation can be modulated by copper, and a copper-modulating strategy suitable for human use is effective in an animal model of tau expression. Translation to human subjects is consequently possible, but justification for such a trial depends on the demonstration that this intervention is functionally important for brain neurons, and will require careful monitoring to avoid adverse effects from copper deficiency.

## Electronic supplementary material

Additional file 1: **Quantification of p35 and p25 bands from Figure S1.** Western blot bands for p35 and p25 were quantified. The mean ± the SEM of the band intensity is represented. Brackets indicate p < 0.05 between the two treatments. (JPEG 830 KB)

Additional file 2: **Iron levels in mouse brain and plasma after zinc treatment.** ICP-MS was used to analyze levels of iron in the brain **(A)** and plasma **(B)**. Metal levels are plotted as parts per billion (ppb) for plasma and μg of metal per gram of homogenized brain tissue. Data is represented as the mean ± SEM of all mice in the treatment group. (JPEG 1 MB)

Additional file 3: **Plot of pTau to total tau for soluble mouse brain fractions following zinc acetate treatment.** Bands from Figure S3 for AT8, AT180, PHF-1 and Tau12 were plotted as a ratio of the phospho-tau species/total tau for each mouse and represented as mean ± SEM of all mice in each treatment group. (JPEG 723 KB)

Additional file 4: **Low concentrations of copper do not affect tau phosphorylation.** Varied copper concentrations were added to the media of SH-SY5Y cells in an effort to clarify whether copper at concentrations below 400 uM affect tau phosphorylation. Western blot bands for phosphorylated tau at T231 show no effect of exogenous copper at concentrations from 5 to 100 uM. (TIFF 745 KB)
