# Supplementary figures and images for: Modulation of tau phosphorylation by environmental copper
Source: Transl Neurodegener. 2014 Nov 17;3:24. doi: 10.1186/2047-9158-3-24 (PMC4322670; doi:10.1186/2047-9158-3-24)

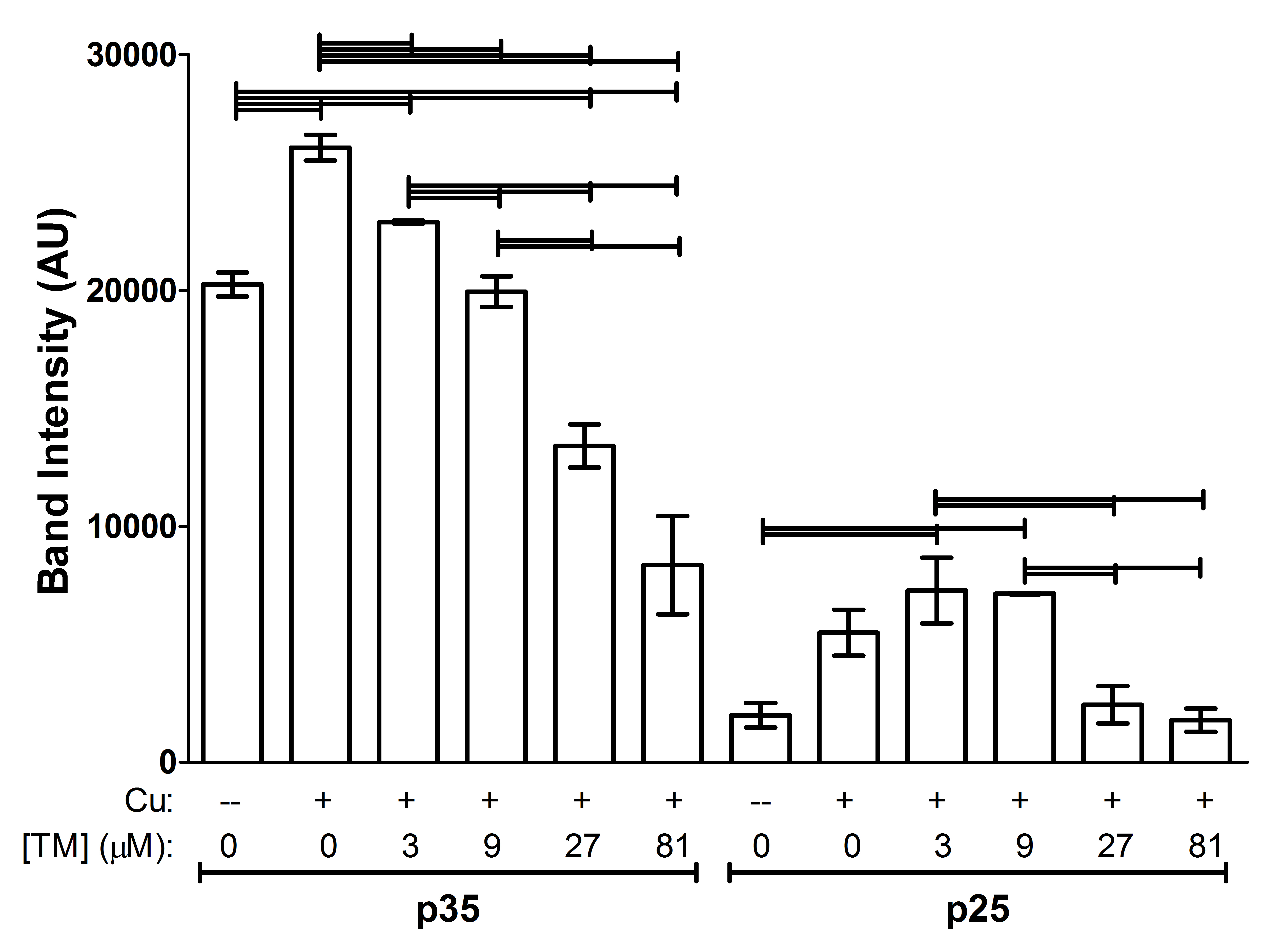

Supplement: Supplementary file 1 — Additional file 1: Quantification of p35 and p25 bands from Figure S1. Western blot bands for p35 and p25 were quantified. The mean ± the SEM of the band intensity is represented. Brackets indicate p < 0.05 between the two treatments. (JPEG 830 KB) [file 40035_2014_72_MOESM1_ESM.jpeg]

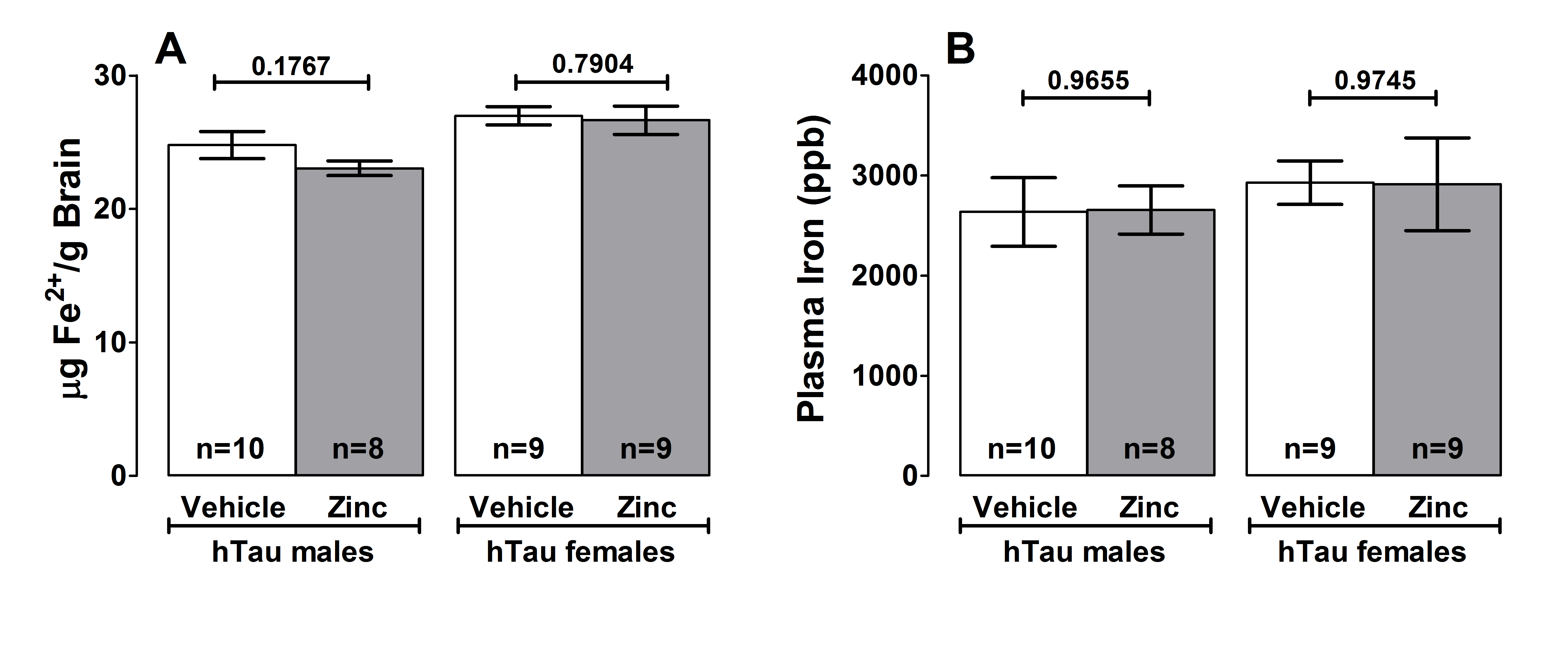

Supplement: Supplementary file 2 — Additional file 2: Iron levels in mouse brain and plasma after zinc treatment. ICP-MS was used to analyze levels of iron in the brain (A) and plasma (B). Metal levels are plotted as parts per billion (ppb) for plasma and μg of metal per gram of homogenized brain tissue. Data is represented as the mean ± SEM of all mice in the treatment group. (JPEG 1 MB) [file 40035_2014_72_MOESM2_ESM.jpeg]

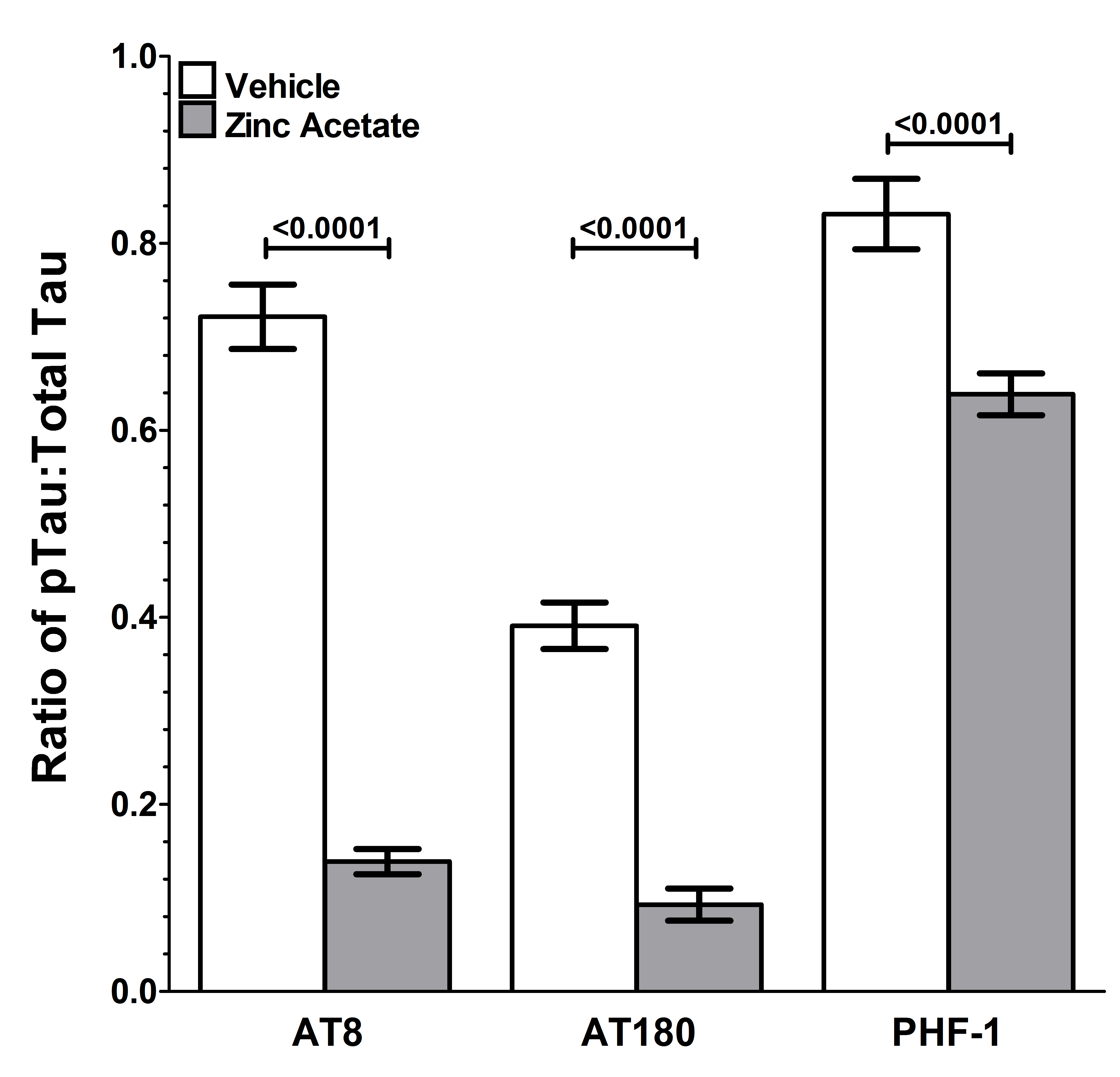

Supplement: Supplementary file 3 — Additional file 3: Plot of pTau to total tau for soluble mouse brain fractions following zinc acetate treatment. Bands from Figure S3 for AT8, AT180, PHF-1 and Tau12 were plotted as a ratio of the phospho-tau species/total tau for each mouse and represented as mean ± SEM of all mice in each treatment group. (JPEG 723 KB) [file 40035_2014_72_MOESM3_ESM.jpeg]

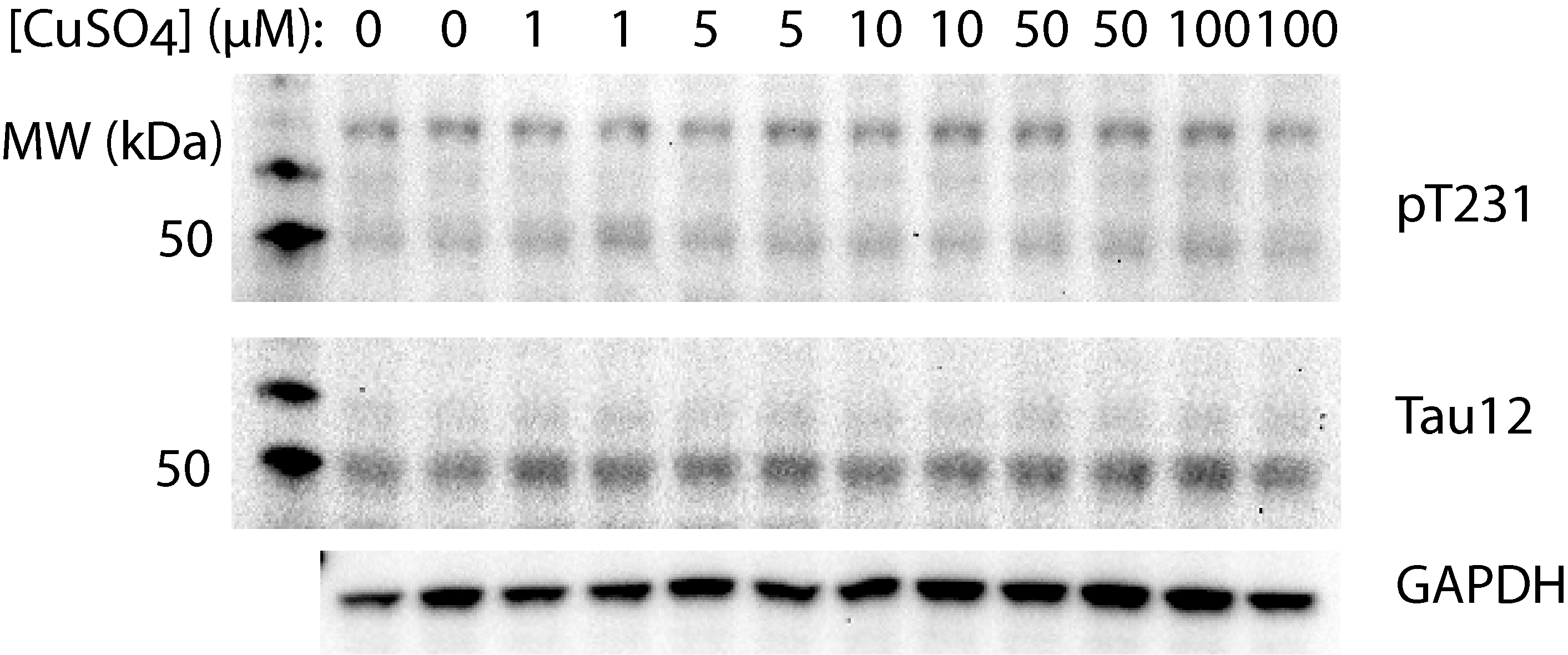

Supplement: Supplementary file 4 — Additional file 4: Low concentrations of copper do not affect tau phosphorylation. Varied copper concentrations were added to the media of SH-SY5Y cells in an effort to clarify whether copper at concentrations below 400 uM affect tau phosphorylation. Western blot bands for phosphorylated tau at T231 show no effect of exogenous copper at concentrations from 5 to 100 uM. (TIFF 745 KB) [file 40035_2014_72_MOESM4_ESM.tiff]
